# Clinical Characteristics of Itch in Renal Transplant Recipients

**DOI:** 10.3389/fmed.2020.615334

**Published:** 2021-01-20

**Authors:** Piotr K. Krajewski, Piotr Olczyk, Magdalena Krajewska, Wojciech Krajewski, Jacek C. Szepietowski

**Affiliations:** ^1^Department of Dermatology, Venereology and Allergology, Wroclaw Medical University, Wroclaw, Poland; ^2^Department of Nephrology and Transplantation Medicine, Wroclaw Medical University, Wroclaw, Poland; ^3^Department of Urology and Oncologic Urology, Wrocław Medical University, Wrocław, Poland

**Keywords:** renal transplant, itch, chronic kidney disease, ESRDCI, renal transplant recipients

## Abstract

**Background:** Chronic itch is the most common symptom in dermatology. End-stage renal disease-associated chronic itch (ESRDCI) is a common burden affecting up to 35% of patients treated with hemodialysis. Kidney transplant (KTx) is believed to be the best renal replacement therapy leading to the elimination of ESRDCI. The study was undertaken to characterize and assess the prevalence of itch among patients after renal transplantation.

**Methods:** Between October 2019 and January 2020, we analyzed the data of 197 patients comprising 121 males (61.4%) and 76 females (38.6%) and aged 54.5 ± 13.6 years. The data collection was performed with a specially designed questionnaire. Level of itch after renal transplantation was assessed with the use of a Numeral Rating Scale, a Visual Rating Scale, and 4-Item Itch Questionnaire. Moreover, the previous 3 days of itching were evaluated.

**Results:** The patients suffered from chronic renal disease for 20.2 ± 12.3 years, with a mean time of pre-transplant dialysis of 2.6 ± 2.4 years and a mean time after the KTx of 8.0 ± 6.5 years. The itch was present in 38.6% of the patients during the hemodialysis, and in 73.7% of cases, the itch ceased completely after the successful transplantation. Moreover, only 2.63% of the cases had no improvement. Nevertheless, the itch was reported in 42 (21.3%) renal transplant recipients (RTR), and in 22 (52.4%) cases, the itch appeared after transplantation. The majority of patients suffering from itch were women (54.8%). Itch in the last 3 days was reported in 21 patients. The itch's severity was assessed with a numerical rating scale (NRS), with the worst severity measured at 6 ± 2.2 points indicating moderate itch. In most cases (57.1%), itch affected multiple body areas. Extremities (50%) and the back (50%) were among the most frequently affected areas. The sensation had a mostly short duration and was most frequently reported in the evening. Only eight patients suffered for the whole day. Hot water was the most frequently reported (16.7%) alleviating factor, whilst sweat was responsible for itch aggravation in 35.9% of cases.

**Conclusion:** Our analysis on representative patients' population indicates that itch after KTx is an important problem. Moreover, it is worth noting that more than half of the RTR did not suffer from itch during dialysis.

## Introduction

Chronic kidney disease (CKD) is recognized as a one of the leading public health problems, affecting up to 13.4% of the global population (1). Moreover, up to 10.6% of people suffer from advanced stages of the disease (stage 3–5) (2). CKD is defined as abnormal renal structure or function with health implications of at least a 3-month duration (1). The diagnostic criteria also include glomerular filtration rate (GFR) < 60 ml/min/1.73m^2^, markers of kidney damage (albuminuria, abnormal urine sedimentation rate, tubular disorders), histologic and radiologic abnormalities, or a history of renal transplantation (3). CKD may be classified in one of the five stages using patients' GFR and one of the three stages based on the patients' albuminuria (2). The loss of kidney function leads to the development of many complications such as anemia, hyperparathyroidism and mineral bone disease, cardiovascular diseases, dyslipidemias, and cancer (4). The disease is associated with frequent and longer hospitalizations, rehospitalization, and premature morbidity (1, 5). One of the associated symptoms of CKD is end-stage renal disease-associated chronic itch (ESRDCI), also known as uremic itch. It is a burdensome symptom that may affect up to 13% of CKD patients (stages 1–5) and up to 35% (lifetime prevalence) of patients treated with hemodialysis (HD) (6). The pathophysiology is not fully understood. Due to the unknown pathomechanism, the treatment of ESRDCI is still a big challenge, and the results are often not satisfactory (7). Kidney transplant (KTx) is considered the best renal replacement therapy; however, its influence on itch was not sufficiently studied (8). The available data suggest that renal transplant recipients (RTR) may suffer from itch less frequently than patients treated with hemodialysis (9–11). Due to the insufficient reports and observations on small groups, we have decided to conduct a study and assess an actual prevalence of itch in RTR. Moreover, we have correlated itch severity with possible pathogenetic factors.

## Materials and Methods

### Study Participants

The studied population consisted of 197 RTR, who are under monthly supervision of the Department of Nephrology and Transplantation Medicine of Wroclaw Medical University. The exclusion criteria included being under the age of 18 years, inability to cooperate and fill out the questionnaire, having a history of chronic dermatological disorders, and having undergone a non-functioning renal transplant. After the inclusion criteria was met by 197 individuals, a medical interview with each patient was performed. Baseline data, including age, sex, BMI, comorbidities, chronic medication, duration of CKD, time of dialysis before KTx, and time after transplantation, were collected.

### Itch Assessment

Itch presence during the whole period after transplantation as well as during the last 3 days was documented. Additionally, localization, the most common aggravating and alleviating factors, specific anti-itch treatments, and usage of emollients were recorded. Moreover, the patients were asked about the presence of itch before and possible improvement after KTx. Itch intensity (worst itch during the last 3 days) was assessed with the following instruments: Numeral Rating Scale (NRS), Verbal Rating Scale (VRS), and 4-Item Itch Questionnaire (4IIQ). Moreover, the patients were asked to evaluate the worst itch intensity during the whole period after KTx. Later, the reported itch was compared to the itch caused by mosquito bites, assessed by RTR with NRS. The VRS is a four-point scale and consists of a list of adjectives describing various levels of symptom intensity: 0 = no itch, 1 = mild itch, 2 = moderate itch, and 3 = severe itch (12). The NRS is comprised of one item and represents the numbers 0 (“no itch”) to 10 (“worst imaginable itch”). The cut-off points for itch NRS are as follows: mild itch (>0 to <3 points), moderate (≥3 to <7 points), severe (≥7 to <9 points), and very severe (≥9 points) (13). 4IIQ was developed and validated by our group some years ago. It assesses not only itch intensity (0–5 points), but also the frequency of itch episodes (0–5 points), areas of affected skin (0–3 points), and sleep disturbance (0–6 points) as a course of chronic itch. The maximum score for this scale is 19 points (14–16).

### Lab Tests Assessments

Results of blood tests conducted periodically on all of the patients, including transplant function (eGFR, creatinine and uremia levels), liver function (ASPAT, ALAT, bilirubin level), calcium and phosphate metabolism (including parathormone levels), hemoglobin, glucose level, and medication level (cyclosporin or tacrolimus), were collected and analyzed in relationship with the presence and severity of itch.

### Statistical Analysis

Statistical analysis was performed using Statistica v. 12 (StatSoft Kraków). The minimum, maximum, mean, and standard deviation numbers were calculated. Analyzed quantitative variables were compared using Mann–Whitney U test and Spearman and Pearson correlations; for qualitative data, the chi-squared test was used. A 2-sided *P* value ≤ 0.05 was considered to be statistically significant.

## Results

### Patients' Characteristics

The group consisted of 197 patients−121 men (61.4%) and 76 women (38.6%). The mean age of the population was 54.5 ± 13.6 years. The group was characterized as slightly overweight with a mean BMI of 26.2 ± 4.4. The majority of patients (88.3%) was treated with three drug immunosuppressive therapies (calcineurin inhibitors, antiproliferative drugs, and glucocorticosteroids [GKS]), while almost every patient (96.4%) was taking GKS. The patients who suffered from CKD for 20.2 ± 12.3 years were treated with hemodialysis for 2.6 ± 2.4 years before RTx and were 7.9 ± 6.5 years after KTx. The majority of RNR suffered from hypertension (161 patients, 81.7%), and 36 subjects suffered from diabetes (18.3%) (Table 1).

**Table 1:** Patients' characteristics.

	**Whole group (*n* = 197)**	**Itch (*n* = 42)**	**No itch (*n* = 155)**
Age in years (mean ± SD)	54.5 ± 13.6	55.7 ± 13.9	54.2 ± 13.6
Sex (Male %)	121 (61.4)	19 (45.2)^*^	102 (65.8)^*^
BMI in kg/m^2^ (mean ± SD)	26.2 ± 4.4	26.4 ± 4.6	26.1 ± 4.3
CKD duration in years (mean ± SD)	20.2 ± 12.3	20.2 ± 11.5	20.2 ± 12.5
Time on HD before KTx in years(mean ± SD)	2.57 ± 2.4	2.2 ± 1.6	2.7 ± 2.6
Time after KTx in years (mean ± SD)	7.9 ± 6.5	8.7 ± 6.9	7.8 ± 6.4
**Immunosuppression**			
GKS (%)	190 (96.5)	38 (90.5)	149 (96.1)
Tacrolimus (%)	174 (88.3)	35 (83.3)	139 (89.7)
Cyclosporine A (%)	38 (19.3)	7 (16.7)	31 (20%)
**Comorbidities**			
Diabetes (%)	36 (18.3)	9 (21.4)	35 (22.6)
Hypertension (%)	161 (81.7)	36 (85.7)	125 (80.7)

* *p = 0.005 (in bold); CKD, chronic kidney disease; SD, standard deviation; BMI, body mass index*.

### Itch Assessment

**Figure 1:**
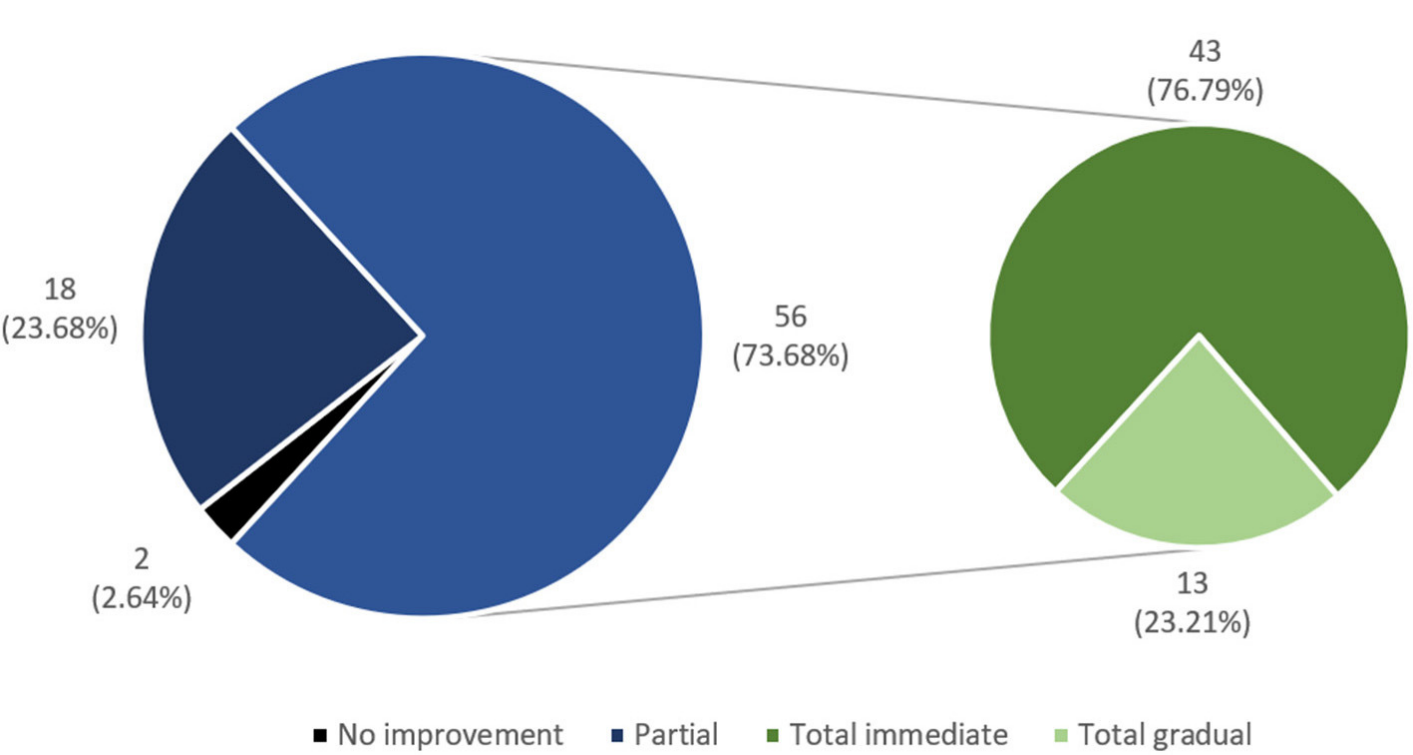
Improvement in itch after kidney transplantation.

**Figure 2:**
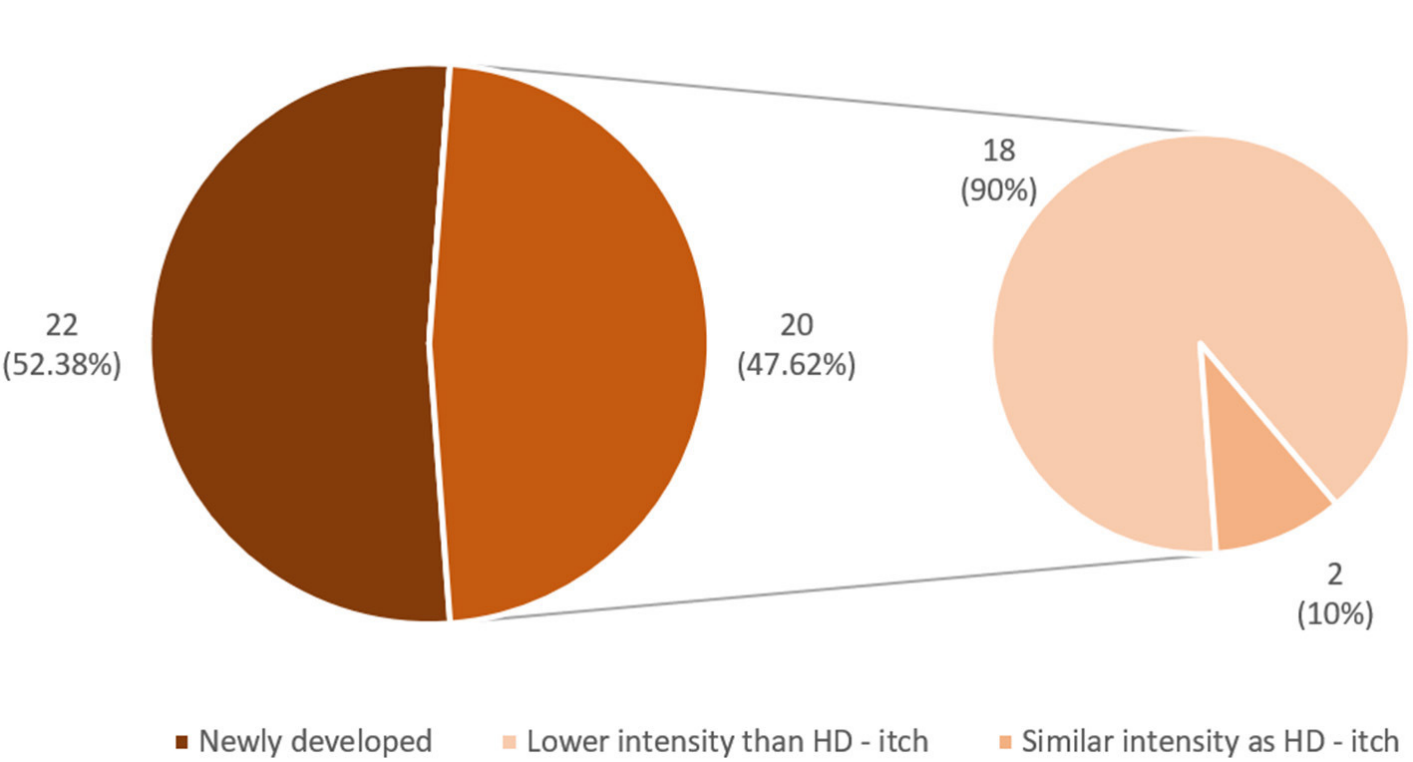
Itch in renal transplant recipients.

**Figure 3:**
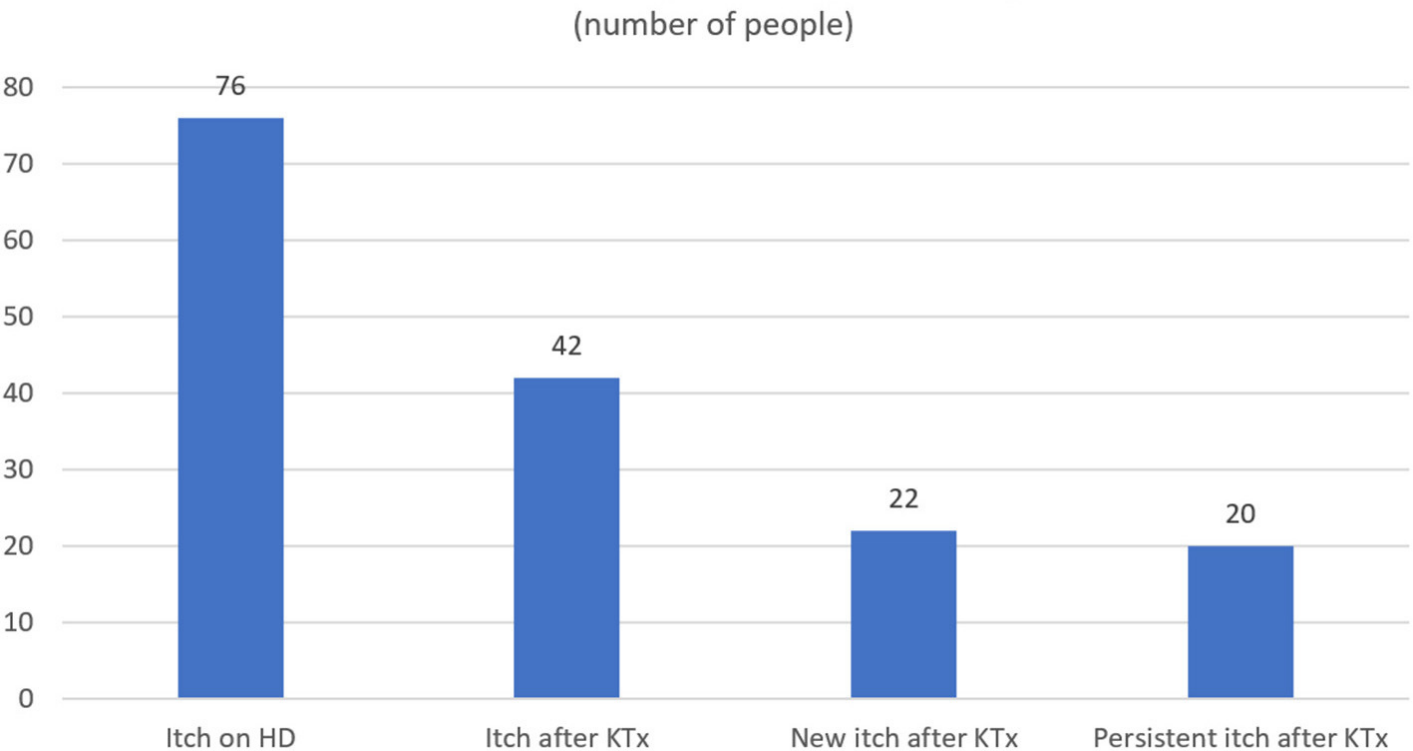
Differences in prevalence of pre- and post-transplantation itch.

Among the studied group of RTR, itch was a common symptom during the period of hemodialysis treatment (76 patients, 38.57%); however, only 42 patients (21.3%) reported itch after KTx. In 56 patients (73.7%), itch disappeared completely after the transplantation. In the majority of them (43 subjects), the relief was instant. In the rest of the subjects, relief was gradual (Figure 1). In the majority of RTR suffering from itch (22 patients, 52.4%), relief appeared after successful transplantation, while the rest (20 patients, 47.6%) reported residual itch from hemodialysis period with lower (18 people) or similar (two people) severity (Figures 2, 3). The WI-NRS itch was at 5.98 ± 2.17 points, which was similar to the itch reported after a mosquito bite (5.36 ± 2.2 points). Among 42 itchy RTR, half (21 people) reported itch in the previous 3 days, and its intensity was assessed as 4.23 ± 1.51 points on WI-NRS. Following the cut-offs for NRS, the majority of patients reported moderate itch (85.7%), two of them mild (9.5%), and only one person was suffering from severe itch (4.8%). According to VRS, 52.38% (11 patients) of RTR who suffered from itch described it as moderate, eight of them (38.1%) described it as mild, and only two (9.52%) described it as severe (Figure 3). Women suffered from itch significantly more than men (*p* = 0.005). Most frequently, the itch affected multiple locations (47.6%), with extremities and back being the most involved (50% of the patients for both locations). In only four patients (9.5%) the sensation was generalized; it affected only one location in the rest of patients (Table 2). Among alleviating factors, patients most often reported very hot and cold water; however, this strategy of relief only helped 16.67 and 14.3% of patients, respectively. The most common aggravating factor was sweat (35.7%) and warm airflow (33.3%). Most frequently, itch occurred in the evening (85.3%) and mostly for a short period of time (85.3%). In eight patients (19%), itch continuously present during the whole day. Only 17 patients (40.5%) were using emollients daily, and four had taken mediation in order to alleviate itching (Table 3). Among possible risk factors, we have found a significant difference (*p* = 0.024) in alkaline phosphatase (ALP) levels between itchy and non-itchy patients (76.1 ± 48.0 and 85.9 ± 33.5 U/l, respectively). There was no correlation found between prevalence (Table 4) or intensity of itch and graft function, the rest of laboratory tests, duration of CKD, time after KTx, time on hemodialysis, and medications administered (detailed data not shown).

**Table 2:** Characteristics of itch in renal transplant recipients.

		**Itchy patients (*n* = 42)**
Sex	Men, *n* (%)	19 (45.2)
	Women, *n* (%)	23 (54.8)
WI-NRS after renal transplantation (mean ± SD)		5.9 ± 2.2
Previous 3-day WI - NRS (mean ± SD) (*n* = 21)		4.2 ± 1.5
Previous 3-day VRS (*n* = 21)	Mild, *n* (%)	8 (38.1)
	Moderate *n* (%)	11 (52.4)
	Severe, *n* (%)	2 (9.5)
	Very severe *n* (%)	0
Itch treatment	Emollients, *n* (%)	17 (40.5)
	Antihistaminic, *n* (%)	4 (9.5)
Localization	Single, *n* (%)	18 (42.9)
	Multiple, *n* (%)	20 (47.6)
	Generalized	4 (9.5)
	Back	21 (50)
	Extremities	21 (50)
	Head	12 (28.6)
	Thorax	7 (16.7)
	Anogenital area	5 (11.9)
	Abdomen	9 (21.4)

*n, number of patients; SD, standard deviation; WI-NRS, Worst Itch-Numeral Rating Scale; VRS, Verbal Rating Scale*.

**Table 3:** Factors responsible for aggravation or alleviation of itch intensity in renal transplant recipients.

**Influencing factor, %**	**Exacerbation**	**Alleviation**	**No impact**
Sleep	14.3	11.9	73.8
Physical activity	7.1	14.3	78.6
Stress	23.8	0.0	76.2
Fatigue	28.6	0.0	71.4
Diet	2.4	0.0	97.6
Very hot water	28.6	16.7	54.8
Cold water	2.4	14.3	83.3
Dry air	33.3	0.0	66.7
Sweating	35.7	2.4	61.9
Cold	2.4	9.5	88.1
Heat	21.4	9.5	69.1

**Table 4:** Differences in laboratory results between itchy and non-itchy patients.

	**Itch (*n* = 42)**	**No itch (*n* = 155)**	***P***
ALAT (U/I)	25.2 ± 16.3	23.8 ± 12.7	0.934
ASPAT (U/I)	26.9 ± 10.1	24.0 ± 8.8	0.127
Billirubin (mg/dl)	0.7 ± 0.3	0.8 ± 0.3	0.218
CRP (mg/l)	6.4 ± 14.2	7.6 ± 15.1	0.845
ALP (U/I)	76.1 ± 48.0	85.9 ± 33.5	**0.024**
GGTP (U/I)	36.0 ± 34.5	45.7 ± 51.3	0.217
Glucose (mg/dl)	106.4 ± 36.5	105.9 ± 33.3	0.913
eGFR (ml/min/m^2^)	55.0 ± 21.0	52.2 ± 16.8	0.780
Creatinine (mg/dl)	1.4 ± 0.5	1.5 ± 0.6	0.136
Uremic acid (mg/dl)	6.6 ± 1.3	8.4 ± 9.3	0.116
Magnesium (mmol/l)	1.8 ± 0.2	1.8 ± 0.2	0.889
Potassium (mmol/l)	4.2 ± 0.3	4.3 ± 0.4	0.180
Calcium (mmol/l)	9.89 ± 0.6	9.9 ± 0.6	0.596
Hb (mg/dl)	13.8 ± 1.7	14.3 ± 1.9	0.719

ALAT, Alanine aminotransferase; ASPAT, Aspartate aminotransferase; CRP, C -reaktive protein; ALP, Alkaline phosphatase; GGT, Gamma-glutamyltransferase; eGFR, Estimated glomerular filtration rate; Hb, Hemoglobin. *p = 0.005.

## Discussion

Chronic itch (CI) is one of the most common symptoms in medicine. The International Forum for the Study of Itch (IFSI) defines it as a sensation that provokes the desire to scratch lasting for at least 6 weeks (17). The actual prevalence of CI is not clear. Studies show that about 8–9% of the adult population experience acute itch, while up to 16.8% experience CI (18). The incidences for both acute and chronic itch seem to increase with age. Acute itch affects up to 60% people in the elderly population every week. The incidence of CI varies among different age groups, being at 12.3% for young adults and even 20.3% for elderly persons. CI may be caused by both dermatological and systemic diseases (17). Among possible systemic causes of chronic itch, authors often mention hepatobiliary diseases, malignancies, infectious diseases, neurological disorders, metabolic diseases, endocrine diseases, and kidney diseases (16, 17, 19, 20). CI poses a high burden and is associated with decrease in quality of life of affected individuals (21).

ESRDCI or uremic itch is defined as CI associated with significant reduction in renal function in patients suffering from CKD. It usually worsens along with decreasing kidney function and may be experienced in up to 35% of individuals with stage 5 CKD treated with hemodialysis (6, 22). Lack of full understanding of pathomechanisms makes treatment difficult and often not fully effective. The frequently reported therapies include drugs such as opioid agonists and antagonists, gabapentin and pregabalin, phototherapy, and antidepressants (7).

The knowledge of the possible effect of KTx on uremic itch is very limited (8) and was reviewed by our group earlier this year. Our study is the first one to evaluate the prevalence of itch on a bigger sample size of RTR. The results of our work confirmed that itch is important burden in RTRs and affects 21.3% of them. The prevalence of itch in our group was different to the studies performed previously. According to the latest study by Schricker et al. (10), only 17% of the patients reported itch, while 12% were diagnosed with CI. Moreover, similarly to our study, authors have found a decrease in the itch prevalence after successful KTx. The mean intensity of recent itch was also lower than in our population (3.2 points and 4.2 points, respectively); however, those results may be different due to the time period taken into consideration (previous 24 h and 3 days, respectively). Correspondingly, Avermete et al. (23) reported only a 2% itch incidence in RTR, and T*ă*ranu et al. (24) reported 5.3%, which was later explained as a drug-induced itch. Moreover, according to Altmeyer et al. (25), both itch and histopathological changes disappeared completely after KTx. In comparison to Panuccio et al. (9), we have reported a much lower itch prevalence (32% of RNRs). Nevertheless, it was still higher than in the healthy controls (11%). Similarly, the incidence of CI was lower than for HD patients; unfortunately, the authors did not mention itch intensity in any group. Our results were similar to those presented by Moloney et al. (26), who reported a 24.9% CI prevalence among 173 RTRs. The burden of CKD-associated itch is well documented (21). The decreased quality of life in RTRs was observed in many studies. Moloney et al. (26) reported that itch had large impact on lives of 57% of kidney recipients suffering from CI. Interestingly, according to Amro et al. (11) RTRs reported a statistically significant clinical decrease in the negative influence on life quality by itching in comparison to the period before transplantation.

The pathogenesis of CKD-associated itch is yet to be fully discovered. However, among possible mechanisms, authors often mention high urea and creatinine blood levels, disturbances in peripheral endogenous opioid system, hyperparathyroidism, neuropathy, xerosis, microinflammation, and neuropathy (27–31). From the most popular risk factors, which have been associated with the pathomechanisms of ESRDCI (7), none have been proven to play a role in the development of itch after successful renal transplant until today. We have correlated possible risk factors with the intensity of itch in RTRs; however, no statistically significant correlation was found. Additionally, no difference in the above-mentioned aspects was found between patients with persistent and newly developed itch. Similar results to our study were obtained by Pannucio et al. (9), who correlated itch intensity and ESRDCI risk factors including inflammation, bone mineral disorders, immunosuppressive therapy, and transplant function. On the other hand, a moderate positive correlation was found between CI intensity and transplant function (*r* = 0.3, *p* = 0.018) in the study by Schricker et al. (10), which was not established in our group. However, we did find a statistically significant difference in itch incidence between sexes (*p* = 0.005). We believe it is due to the different itch perception in modulation in women, as shown in the paper by Stumpf et al. (32). Interestingly, we have found significantly decreased levels of ALP in patients with itch. It is well documented that increased ALP may be a sign of hepatobiliary disorders, including cholestasis and hepatic cirrhosis (33). Those disorders are strongly correlated with high itch prevalence in those patients (34). On the other hand, low ALP levels have never been associated with incidence of itch. Although the patomechanism of itch in RNR is not clear, it is most probably multifactorial and the polypharmacy in those patients could contribute to the development and intensity of itch. Regarding the most frequent exacerbating factors, our patients reported sweat and warm airflow. These factors are similar to those present in atopic dermatitis or psoriasis (14, 35). Regarding sweat, there are multiple mechanisms that may induce or aggravate itch (e.g., abnormality in sweat components or “sweat allergy”). On the other hand, itch exacerbations due to warm air may be produced by abnormal hyperesthesia of the RNRs' skin. The sensitization of peripheral nerve may cause patients to feel thermal stimulation as itch (35).

We understand that our study has some limitations. Due to the lack of reports on severity of itch during hemodialysis in our patients, we could not assess an actual decrease in itch. However, we believe that because of the long period of time (7.9 ± 6.5 years) following KTx, the reported severity might be biased. There is no agreement on the role of the dry skin in the pathogenesis of ESRDCI (36). Therefore, in this project, we have not analyzed the relationship of itch prevalence or intensity to dryness of the skin. Moreover, there was no possibility to assess atopic predisposition. As there are almost no studies on itch in RNR, in this project we concentrated on incidence and intensity of itch in this group of subjects, and therefore we did not assess the burden of itch. We are aware that the assessment of itch's consequences, including quality of life impairment, will add value to the field. Additionally, it is important to remember that every patient in our group was taking calcineurin inhibitors, either cyclosporine A or tacrolimus. The treatment, besides its anti-rejection mechanisms, may have influenced the incidence and severity of itch. Both of the drugs were proven to be effective as anti-itch agents in animals (37), and cyclosporine A was effective in patients with prurigo nodularis and lichen planus (38, 39). Nevertheless, we have not observed a difference in prevalence and intensity of itch between different calcineurin inhibitors, and it is not possible to perform a study within RTRs without one of these two drugs involved.

In conclusion, to the best of our knowledge, this is the biggest study assessing the prevalence and intensity of itch in RTRs. It is also one of the few studies that correlated itch with common uremic itch risk factors. We have shown and confirmed that successful KTx may be of benefit in the treatment of ESRDCI. Nevertheless, it is worth noting that the majority of itch in RNR was developed after transplantation. The pathomechanism is still unknown, and future studies on representative samples are necessary to make this topic clearer.

## Data Availability Statement

The raw data supporting the conclusions of this article will be made available from the authors on request.

## Ethics Statement

The studies involving human participants were reviewed and approved by Bioethics committee of Wroclaw Medical University. The patients/participants provided their written informed consent to participate in this study.

## Author Contributions

PK: research concept and design, collection and/or assembly of data, data analysis and interpretation, writing the article, and final approval of article. PO: collection and/or assembly of data, data analysis and interpretation, and writing the article. MK and WK: research concept and design, data analysis and interpretation, writing the article, and critical revision of the article. JS: research concept and design, data analysis and interpretation, writing the article, critical revision of the article, and final approval of article. All authors contributed to the article and approved the submitted version.

## Conflict of Interest

The authors declare that the research was conducted in the absence of any commercial or financial relationships that could be construed as a potential conflict of interest.
